# Lycopene as a Natural Antioxidant Used to Prevent Human Health Disorders

**DOI:** 10.3390/antiox9080706

**Published:** 2020-08-04

**Authors:** Muhammad Imran, Fereshteh Ghorat, Iahtisham Ul-Haq, Habib Ur-Rehman, Farhan Aslam, Mojtaba Heydari, Mohammad Ali Shariati, Eleonora Okuskhanova, Zhanibek Yessimbekov, Muthu Thiruvengadam, Mohammad Hashem Hashempur, Maksim Rebezov

**Affiliations:** 1 University Institute of Diet and Nutritional Sciences, Faculty of Allied Health Sciences, The University of Lahore, Lahore 54000, Pakistan; muhammad.imran8@dnsc.uol.edu.pk; 2 Non-Communicable Diseases Research Center, Sabzevar University of Medical Sciences, Sabzevar 9617913112, Iran; f-ghorat@alumnus.tums.ac.ir; 3 Department of Diet and Nutritional Sciences, Faculty of Health and Allied Sciences, Imperial College of Business Studies, Lahore 53720, Pakistan; hod.ddns@imperial.edu.pk; 4 Department of Clinical Nutrition, NUR International University, Lahore 54000, Pakistan; habib.rehman@niu.edu.pk; 5 Department of Food Science and Human Nutrition, University of Veterinary and Animal Sciences, Lahore Syed Abdul Qadir Jillani (Out Fall) Road, Lahore 54000, Pakistan; foodandnutrition1983@gmail.com; 6 Poostchi Ophthalmology Research Center, Shiraz University of Medical Sciences, Shiraz 7134845794, Iran; mheydari@sums.ac.ir; 7 Department of Technology of Food Products, K.G. Razumovsky Moscow State University of Technologies and Management (the First Cossack University), 109004 Moscow, Russia; m.ali.sh@semgu.kz; 8 Food Science and Technology Department, Shakarim State University of Semey, Semey 071412, Kazakhstan; eokuskhanova@gmail.com (E.O.); zyessimbekov@semgu.kz (Z.Y.); 9 Department of Crop Science, College of Sanghuh Life Science, Konkuk University, Seoul 05029, Korea; 10 Noncommunicable Diseases Research Center, Fasa University of Medical Sciences, Fasa 7461686688, Iran; 11 Department of Persian Medicine, Fasa University of Medical Sciences, Fasa 7461686688, Iran; 12 V.M. Gorbatov Federal Research Center for Food Systems of Russian Academy of Sciences, Moscow 109029, Russia; rebezov@ya.ru; 13 K.G. Razumovsky Moscow State University of Technologies and Management (the First Cossack University), Moscow 109004, Russia

**Keywords:** lycopene, antioxidants, oxidative stress, cancer, diabetes, cardiovascular diseases, skin disorders

## Abstract

Lycopene, belonging to the carotenoids, is a tetraterpene compound abundantly found in tomato and tomato-based products. It is fundamentally recognized as a potent antioxidant and a non-pro-vitamin A carotenoid. Lycopene has been found to be efficient in ameliorating cancer insurgences, diabetes mellitus, cardiac complications, oxidative stress-mediated malfunctions, inflammatory events, skin and bone diseases, hepatic, neural and reproductive disorders. This review summarizes information regarding its sources and uses amongst different societies, its biochemistry aspects, and the potential utilization of lycopene and possible mechanisms involved in alleviating the abovementioned disorders. Furthermore, future directions with the possible use of this nutraceutical against lifestyle-related disorders are emphasized. Its protective effects against recommended doses of toxic agents and toxicity and safety are also discussed.

## 1. Introduction

Lycopene is a phytochemical mainly found in tomato and tomato-based products. It is a tetraterpene compound consisting of eight isoprene units and 11 double linear bonds. Lycopene is a non-pro-vitamin A carotenoid [1,2]. However, it is mentioned as an intermediate of carotenoid synthesis in plants [3]. Some fruits’ and vegetables’ red and orange coloration is attributed to this liposoluble pigment [2]. Despite this, it is found in some non-red or non-orange plants, such as asparagus and parsley [4]. It should be noted that lycopene cannot be synthesized in the human body. Therefore, it must be consumed in a daily diet [5]. The absorbed lycopene is mostly stored in the liver, adrenals, and prostate. Moreover, it can be found in other body parts (e.g., brain and skin) in a lower concentration [6]. Lycopene bioavailability can be decreased by ageing, and some of the pathological states, such as cardiovascular diseases (CVDs) [7]. Therefore, its supplementation—through various means, such as pasteurized watermelon juice—has been suggested to increase its circulating serum level in populations in need [8,9].

Interestingly, carotenoids have the highest concentration in the serum and tissues between different natural antioxidants. In addition, according to one study on a United States population, lycopene is the leading carotenoid in plasma and tissues [10]. Furthermore, second to astaxanthin (amongst different carotenoids), lycopene is the most potent antioxidant. It is an important deactivator of reactive oxygen species (ROS). For instance, it can remove singlet oxygen two and ten times more than beta-carotene and alpha-tocopherol, respectively [11]. In recent years, there has been an ever-increasing interest in lycopene’s health benefits. It is not only a potent antioxidant; its beneficial effects in the prevention and treatment of a wide variety of diseases have been assessed and approved by many systematic reviews and meta-analysis studies [12].

For instance, it has been shown that a higher dietary intake and circulating concentration of lycopene have protective effects against prostate cancer (PCa), in a dose-dependent way [13]. The findings approve this effect for the whole tomato, cooked tomato, and sauces consumption [14]. In addition, lycopene’s inhibitory effect on the proliferation and progression of the colorectal cancer cells has been investigated [15]. Moreover, the protective role of lycopene in metabolic syndrome has been well-studied. It seems that different metabolic syndrome components can be improved by lycopene supplementation “*rather than demonstrating consistent improvement in a single component*” [16]. Lycopene can be used in different CVDs, too [17]. It can improve ventricular remodeling, and vascular and endothelial function. In addition, it is useful in the reduction in atherosclerotic plaque size, and arterial stiffness. Therefore, it can be concluded that this nutraceutical has a vital role in the primary and secondary prevention of CVDs [3]. In addition, lycopene is a natural neuroprotective agent. It seems that this carotenoid contributes to cognitive longevity [18] and the treatment of several neuronal diseases, including cerebral ischemia, Parkinson’s disease (PD), Alzheimer’s disease (AD), subarachnoid haemorrhage, epilepsy, Huntington’s disease, and depression [19]. There are several other clinical applications for this available phytochemical. It can be used in different skin [20], oral, and dental diseases, too [21]. This paper aims to make a comprehensive review on lycopene, from its sources and uses amongst different societies, to its biochemistry and its different biological effects. Furthermore, its protective effect against various toxins, its recommended dose, and toxicity and side effects were discussed separately.

## 2. Natural Sources of Lycopene and Its Use Amongst Different Societies

Tomato and tomato-based products are the major dietary sources of lycopene and account for approximately 80% of the consumption of lycopene in western countries. It is also present in a high amount in watermelon, guava, pink grapefruit, rosehips, papaya, and apricot [22]. Table 1 shows its amount in different sources [23,24,25]. Lycopene contents significantly differ in diverse varieties of tomato and other fruits and vegetables. Its content depends on several factors: 1, the degree of maturity; 2, the weather temperature (lycopene is transformed to beta-carotene at temperatures higher than 35 °C); and 3, the soil quality (conditioners including necessary microorganisms may cause lycopene content to be increased about 36%) [26].

Lycopene intake is also varied among different people in different regions [25]. In the population of European countries, lycopene consumption from natural sources is varied from approximately 0.5 to 5 mg/day. Nevertheless, variation in lycopene consumption was reported, where adults in Spain consume less lycopene (1.64 mg/day) compared to adults from France, and the UK, where the intake was from 4.43 to 5.01 mg/day [27,28]. In the United States, daily intake is more than 7 mg/day [29]. Higher utilization of fruits and vegetables, especially tomato-based products, may result in occasional intakes of 20 mg lycopene/day. Approximately 50–65% of the total exposure to lycopene was produced from natural sources. Some of the important daily sources of lycopene are tomatoes, soups, pasta dishes, tomato sauces and ketchup [30].

## 3. Biochemistry of Lycopene

In nature, there are more than 600 carotenoids which are mostly colored, produced by plants, fungi and bacteria. Carotenoids have two main groups: (i) the highly unsaturated hydrocarbons (α, β-, and γ-carotene, lycopene), (ii) xanthophylls (lutein, β-cryptoxanthin, and zeaxanthin). Xanthophylls possess a minimum of an oxygenated group on their end rings, while unsaturated hydrocarbon carotenoids contain just carbon and hydrogen atoms with no oxygen [31]. Modified carotenoid structures, such as the cyclization of terminal groups and inserting oxygen, are linked with diverse forms of carotenoids resulting in color alterations and also various antioxidant properties [32].

Lycopene (C40H56), as a hydrocarbon carotenoid, includes an acyclic open chain structure containing 13 double bonds that are subjected to isomerization and differed *cis* isomers, such as 5, 9, 13, and 15 observed in plants and blood plasma [33]. Naturally, lycopene can be found in all the *trans* isomers, but the *cis* isomers are the most typical type in tissue and plasma [34]. This takes place during food pretreatment, preparation, processing, storage and transportation, as well as during metabolism in the body [35]. The conversion of *cis* to *trans* isomerization takes place in enterocytes, liver and stomach [36,37]. The absorption of lycopene in the intestine is aided by scavenger receptor CD36 and B1 [38,39]. Partial metabolism can be seen in the enterocyte assisted by two enzymes: 15′-oxygenase-1, β-carotene 15, which is linked with blood lycopene level, and 10′ oxygenase-2, β-carotene-9 [40,41].

**Figure 1:**
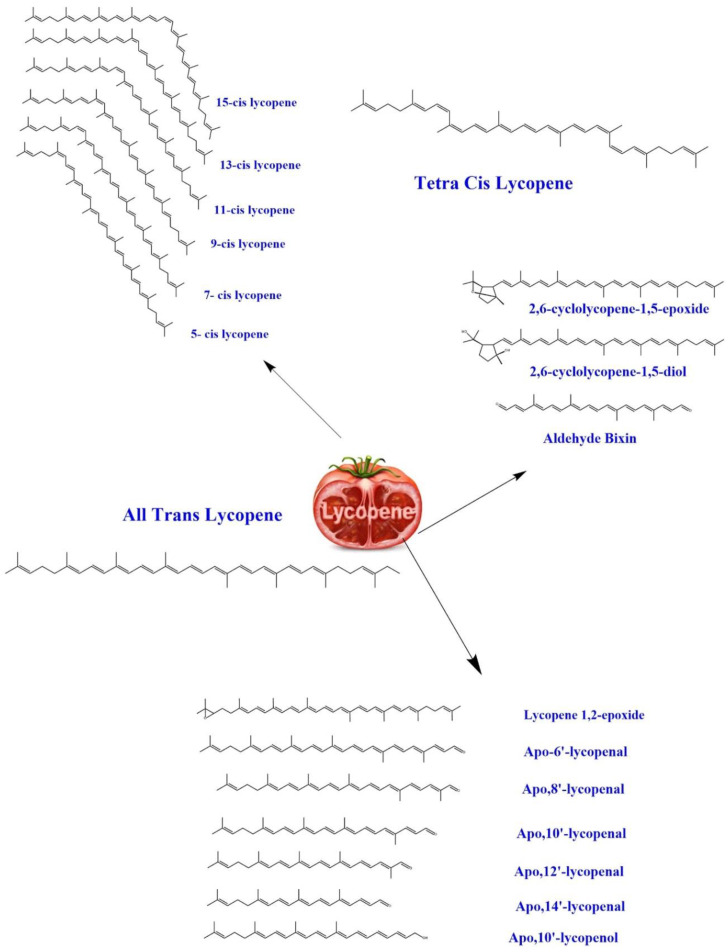
The molecular structures of lycopene and its derivatives and isomers.

Furthermore, there is limited research on labelled lycopene molecules. For instance, a study conducted by Ross et al. on 14C-labelled lycopene (92% *trans*-lycopene) revealed that the *trans*-lycopene was completely isomerized (5-*cis*, 9-*cis*, 13-*cis*, and 15-*cis* lycopene isomers) following dosing and quickly metabolized into polar metabolites entered in the urine [42]. The quick exclusion of 14CO_2_ indicated sufficient oxidization of the ingested lycopene. In the compartmental model research, 13C-labelled lycopene was used. No significant differences were found in the bioavailability of *cis*- and *trans*-lycopene (24.5 vs. 23.2%, respectively). Moreover, it was confirmed that post-absorptive *trans*-to-*cis* isomerization affects tissue and plasma isomeric profiles [43]. The most thermodynamical lycopene firm configuration has shown the all-*trans*-isomer. Light, heat, or many chemical reactions may cause isomerization from the *trans*-isomer toward several mono- or poly-*cis* types, of which two types are non-conjugated. In comparison, 11 types are conjugated double bonds, forming a chromophore involved in visible ruby color, as well as lycopene antioxidant activities [23]. Figure 1 shows the molecular structures of lycopene and its derivatives and isomers.

## 4. Biological Effects

There is a wide variety of pharmacological actions and clinical applications which have been attributed to the lycopene by a chain of researches worldwide. Table 2 summarizes these insights by discussing different mechanisms of action briefly.

### 4.1. Anticancer

Inflammation is known as one of the most important key points in cancer. Therefore, lycopene, as one of the most potent antiinflammatory nutraceuticals, is under research in many preclinical and clinical cancer studies. Epidemiological studies showed a reverse relation between its serum level and cancer occurrence [80]. In addition, increased consumption of lycopene (from each source) has been reported to be associated with a decreased risk of a wide variety of cancers, such as breast, lung, ovary, prostate, stomach, and ovary [81].

In laying hens, supplementation (200 to 400 mg lycopene/kg diet) significantly reduced ovarian tumor occurrence, and also the tumors’ size and number. Moreover, supplementation with lycopene reduced the STAT3 expression in ovarian tissues via the induction of the protein inhibitor of activated STAT3 expression [44,45]. The lycopene extract (dose: 400 and 800 μg/mL) reduced the risk of human breast adenocarcinoma cells (MCF-7). It could modify the mitochondrial membrane potential, DNA fragmentation and also affected the granularity and size of the MCF-7 cell. Additionally, it does not result in noticeable damage to the cell membrane, necrosis and regular apoptosis [46,47]. Treatment of HGC-27 cells with lycopene exhibited significant improvement in LC3-I, p-ERK proteins expressions [48].

Moreover, in vitro and in vivo investigations exhibited that lycopene (dose: 0.1–5 μM) affected human liver adenocarcinoma (SK-Hep-1) cells and indicated a substantial reduction in NOX activity. Moreover, it inhibits the protein expression of NOX4, NOX4 mRNA and ROS intracellular amounts. The transforming growth factor-β (TGF-β)-related effects were antagonized via the lycopene (2.5 μM) incubation with SK-Hep-1 cells. Through small interfering RNA (siRNA) transient transfection against NOX4, the results reveal that NOX4 downregulation could mimic lycopene via the inhibition of cell migration and MMP-9 and MMP-2 activities in incubating with/without TGF-β on SK-Hep-1 cells [49].

In another study, lycopene consumption markedly decreased the metastatic load in a tumor-bearing rat model of ovarian carcinoma. Lycopene has anti-tumorigenic effects on paclitaxel and carboplatin. Its consumption further inhibits biomarkers for ovarian cancer, such as *CA125* expression. The anti-proliferative and anti-metastatic impacts were complemented via *ITGB1*, *MMP9*, *ITGA5*, *FAK*, *ILK* and *EMT* markers’ down-regulated expression, decreasing the MAPK activity and inhibiting integrin α5 protein expression [50].

*FOXO3a* is crucial to modify cell death genes expression. Treatment with lycopene reduced cell hyperproliferation induced by UVB and ultimately promoted apoptosis and reduced CDK2 and CDK4 complex in SKH-1 hairless mice and human keratinocytes. It is sequestered in cytoplasm and can phosphorylate against UVB irradiation, although pretreatment with lycopene reduced all these activities. *FOXO3a* gene ablation reduced lycopene-caused attenuation in cell hyper-proliferation, CDK2, and CDK4 complex. *FOXO3a* siRNA transfection prevents lycopene-related elevation in cell apoptosis, cleaved PARP expression and BAX. Furthermore, protein kinase B (AKT) loss can induce more lycopene-caused *FOXO3a* dephosphorylation, whereas losing the mechanistic target of rapamycin complex 2 (mTORC2) via transfection with a rapamycin-insensitive companion of mammalian target of rapamycin (RICTOR) siRNA induces AKT phosphorylation to amounts like the levels gained by lycopene. On the other hand, AKT/mTORC2 overexpression decreases the lycopene impact on the *FOXO3a* expression and AKT phosphorylation, indicating the link between lycopene and the mTORC2/AKT signaling negative modulation [82]. Interestingly, lycopene treatment for human breast carcinoma cell line MCF-7 in vitro, leading to cell shrinkage and breakage, is dependent on the dose and time. Moreover, lycopene treatment up-regulates Bax mRNAs and p53 expression [83].

Lycopene doses of 0, 10, 20, and 30 µM were used to treat human colorectal cancer cells. Lycopene’s effect on cell proliferation was evaluated through the 3-(4,5-dimethylthiazol-2-yl)-2,5-diphenyl tetrazolium bromide (MTT) method. Prostaglandin E2 (PGE2), and NO levels declined after lycopene administration [84].

Treatment of human PCa cells was done with lycopene extracts (5 mg/mL) and indicated a significant drop in cell viability and apoptotic cell population [85]. In addition, lycopene nanoparticles (in lipid-based wall material in MCF-7 cells), in a time- and concentration-dependent fashion, significantly reduced cell viability and cell survival [86]. Regarding human trails, in head and neck squamous cell carcinoma, a lycopene concentration of >10 µM for a period of 24 h prevents Cal27 and FaDu cell growth, dependent on the dose and time. Lycopene doses of 25 µM decrease invasion abilities and have significant inhibitory effects. Moreover, lycopene consumption up-regulates B cell lymphoma linked with X protein, a pro-apoptotic protein, and results in inhibiting mitogen-activated and B-protein kinase signaling route [87,88].

In another study on PCa, lycopene, or its metabolite (i.e., apo-10-lycopene), enhanced β, β-carotene-9′, 10′-oxygenase 2 (BCO2) expression, while decreasing cell proliferation in androgen-sensitive cells. However, lycopene cannot modify BCO2 expression and cell development in androgen-resistant cells. The mutant (enzymatically inactive)/wild-type BCO2 exogenous expression in PCa cells decreased NF-κB function, DNA binding and reduced NF-κB translocation in the nucleus [89]. In hepatocellular carcinoma, lycopene supplementation (lycopene dose: 6.6 mg/kg BW/day) inhibited NNK-related α7 nicotinic acetylcholine receptor expression (lung), NF-κB and CYP2E1 (liver) and reduced NNK-related death and pathological abrasions in ferrets’ livers and lungs [90]. However, cell invasion, proliferation and migration showed no alteration in a dose-dependent fashion. In another research, lycopene intake is protective against PCa in 46,719 men. It seems that this effect is via decreasing the gene fusion transmembrane protease, serine 2 (TMPRSS2): v-ets avian erythroblastosis virus E26 oncogene homolog (ERG) [91].

### 4.2. Antidiabetic

There is scientific evidence which supports the beneficial role of lycopene against diabetes. Regarding animal studies and epidemiological surveys, it can be used for both the prevention and treatment of diabetes [92]. In a diabetic rat model (streptozotocin (STZ)-induced), lycopene decreased diabetes-associated pancreas injury and urine and blood glucose levels. In addition, it increased serum insulin levels [93]. The interactions between advanced glycation end products (AGEs) and their receptors (RAGEs) induce oxidative stress-like conditions. Moreover, in vitro and in vivo investigations revealed that lycopene slows down ribose-related forming of AGE in HK-2 cells, while in vivo studies showed that in kidneys, it down-regulates RAGE expression. HK-2 cells with reduced amounts of RAGE indicated reduced NF-κB and MMP 2 expressions [56]. Lycopene supplementation significantly reduced serum nitrate–nitrite levels in diabetic Wistar rats [57].

In addition, lycopene exhibited some effects on histological alterations and also Bcl-2 family gene expression in the STZ-induced diabetic rats’ hippocampus. Lycopene (dose: 4 mg/day/kg) decreased the Bax expression. However, it improved Bcl-xL amounts and Bcl-2 [55].

In addition, lycopene considerably controlled high-fat diet (HFD)-related elevation of glucose, insulin level, fasting blood glucose, insulin intolerance and decrease hepatic glycogen level. Lycopene decreases the fatty acid synthase (FAS), sterol regulatory element-binding protein 1c (SREBP-1c), and Acetyl-CoA carboxylase (ACC1) expression in HFD mice. The consumption of lycopene considerably decreases the phosphorylation and STAT3 expression in livers in HFD mice. The adenovirus treatment with lycopene noticeably inhibited a reduction in SREBP-1c expression. Moreover, an increase in STAT3 motioning via adenovirus noticeably blocked a decrease in fasting blood insulin as well as glucose amount [94].

### 4.3. Cardioprotective

Lycopene is a cardioprotective nutraceutical. Different research showed a protective effect against atherosclerosis and several CVDs [58,95]. It can scavenge some of the potent oxidants that are known to be associated with atherosclerosis. Moreover, lycopene reduces oxidation of cholesterols. Therefore, the early steps in atherosclerosis are prohibited [96]. In hypercholesteremic rats, lycopene administration (50 mg/kg) significantly decreased low-density lipoprotein-cholesterol (LDL), total cholesterol (TC), thiobarbituric acid reactive substances (TBARS), very low-density lipoprotein-cholesterol (VLDL), triglycerides (TG), and increased high-density lipoprotein-cholesterol (HDL) level [59,97]. Furthermore, lycopene was studied for its positive health benefits on some important inflammatory factors (e.g., CRP, IL-6), lowering blood pressure, pulse wave velocity, adhesion molecules (ICAM-1) and endothelial function (flow-mediated dilation) [60]. Moreover, lycopene was found to protect grafted vessels by regulating the expression of key proteins which are essential in arteriosclerosis. It remarkably lessened the expression of ROCK1, Ki-67, ICAM-1 and ROCK2, whereas it improved the expression of eNOS-implanted arteries and cGMP plasma concentration. In summary, lycopene is able to improve the vascular arteriosclerosis in the case of allograft transplantation by downregulating the expression of Rho-related kinases and by regulation of the NO/cGMP pathways expression, as well [61].

Importantly, lycopene consumption protects the heart from Atrazine (ATZ) exposure and also prevents ATZ-induced damage [98]. The results reveal that oral intake of lycopene also prevents myocardial ischemia-reperfusion (I/R) injury. Lycopene use (1 μM) prior to the incidence of re-oxygenation significantly prevents hypoxia/reoxygenation (H/R)-induced cardiomyocyte death. Lycopene (1 μM, intravenous) considerably inhibited myocardial infarction (MI), production of ROS and c-Jun *N*-terminal Kinase (JNK) phosphorylation in I/R mice [7,99]. Moreover, lycopene has a protective effect against the apoptosis mediated by endoplasmic reticulum stress (ERS), which is due to H/R in H9C2 cardiomyocytes. In I/R myocardium, lycopene dose (10 μM) diminished the lactic dehydrogenase, apoptosis ratio and protein expression of 78 kDa glucose-regulated protein (GRP78). It can be associated with phosphorylated JNK (pJNK), C/EBP homologous protein (CHOP) and Caspase-12 routes [100].

It also improved ERS caused by apoptosis that can be observed via decreasing the expression of CHOP/growth arrest and DNA damage-inducible gene (GADD153), Bax/Bcl-2, caspase-3, caspase-12 activity in H/R-treated cardiomyocytes. It was also reported that lycopene is capable of preventing thapsigargin (THG)-related ERS through a reduction in the GRP78 and CHOP/GADD153 protein expression in the THG group, considerably enhancing cell viability which is caused by THG. It also inhibited apoptosis in THG-treated cardiomyocytes [35,101,102]. Another study reported that lycopene (dose: 10 mg/day/kg) in mice with post-MI ventricular remodeling notably reduced the expression of TGF-β1, collagen III, collagen I, IL-1β, TNF-α, caspase-3, caspase-8 and caspase-9, and reduced NF-κB activity. The administration of lycopene also decreased the level of ventricular remodeling post-MI [103]. Moreover, lycopene supplementation improves the endothelial function in patients with CVD diseases [104].

### 4.4. Antioxidative

Lycopene is a well-known antioxidant. It can protect DNA, proteins, and lipids against oxidation. In addition, “*lycopene can act on other free radicals such as hydrogen peroxide, nitrogen dioxide and hydroxyl radicals*” [105].

Oxidative stress, as well as inflammation, is essential in acute pancreatitis (AP) pathogenesis. Lycopene (50 mg/kg) significantly prevented AP by a substantial decrease in MPO activity, TNF-α and down-regulating *iNOS* gene expression along with decreasing NO level, increasing pancreatic glutathione (GSH), decreasing serum α-amylase and lipase functions in Wistar rats [64]. Fluorosis can induce oxidative stress by activating MAPK cascade that can cause cell apoptosis. The combination of vitamin E and lycopene prevented fluoride-induced spermatogenic cell apoptosis in rats. Both decreased the expression of rescued clustering and toxicity induced by fluorosis. As well as this, it also decreased the improved JNK and also phosphorylation of the ERK [106].

Another study showed the lycopene role via down-regulating the expression rate of the proprotein convertase subtilisin/kexin type-9 (PCSK-9) by inhibiting hepatocyte nuclear factor-1α, improving LDL-receptor, and sterol regulatory element-binding protein-2. Lycopene reduces Apo-CIII to assemble with lipoprotein lipase (LPL) bonding, too. Likewise, lycopene improved LPS-induced oxidative stress by increasing total antioxidant and HDL-related PON-1 function as well as down-regulating the plasma level and inflammatory mediators expression [107].

Interestingly, lycopene intake (50 mg/kg BW/day) improved d-galactose in CD-1 male mice having cognitive defects. It also improved histopathological injury and repaired brain-derived neurotrophic factor (BDNF) amounts in mice’s hippocampal area. Lycopene considerably increased antioxidant enzymatic activity and decreased inflammatory cytokines in d-galactose-administrated mice serum. Furthermore, lycopene supplementation improves mRNA expressions of the NQO-1 and HO-1 as antioxidant enzymes. It was also found to downregulate inflammatory cytokines (i.e., TNF-α, and IL-1β) in the hippocampus of the mice. Lycopene noticeably improves the *GFAP* and Iba-1 expression as the glial cells’ inflammatory makers. Lycopene decreased neuronal oxidative damage by activating Nrf2, as well as by inactivating NF-κB translocation in H_2_O_2_-related SH-SY5Y cell model [65]. Moreover, in a rat model of colitis, it significantly reduced the malondialdehyde (MDA), overall sialic acid, DNA fragmentation concentration, and improved the function of the antioxidant enzyme [108]. In addition, lycopene has significant effects against pathological findings caused by carbofuran on oxidative and biochemical stress biomarkers. It (18 mg/kg BW) markedly improved the serum acetylcholinesterase, albumin, protein and lipids. Furthermore, it improved the catalase (CAT), superoxide dismutase (SOD), and GSH levels, and antioxidant capacity [109].

Lycopene administration considerably improved cognitive defects, noticeably reduced MDA levels and elevated GSH-Px activity, and remarkably reduced tau (or τ proteins) hyperphosphorylation at Thr231/Ser235, Ser262, and Ser396 in P301L transgenic mice’s brains [109] (104). In a study conducted on an animal model, in which male mice (C57BL/6) were orally administrated (lycopene dose; 10 or 100 mg/kg), the results reveal that lycopene significantly reduced ROS synthesis in SK-Hep-1 cells while inhibiting NADPH oxidase, which was carried by the protein kinase C (PKC) pathway. Lycopene was also found to be effective against hepatotoxicity by acting as an antioxidant, regulating total glutathione (tGSH) and CAT concentrations, reducing glutathione disulfide (GSSG) and oxidative damage via reducing protein carbonylation. It also elevated MMP-2 down-regulation [110]. Regarding regulator of calcineurin 1 (RCAN1), lycopene reduced mitochondrial ROS and intracellular concentrations, NF-κB activity, Nucling expression, while decreasing the respiration per mitochondrion, MMP and glycolytic activity in RCAN1-overexpressing cells. It suppressed cell death, caspase-3 activation, DNA fragmentation, and cytochrome-c secretion in RCAN1-overexpressing cell lines [111].

The antioxidative role of lycopene was tested in the field of nephrology, also. In gentamicin-induced nephrotoxicity, intake of lycopene and rosmarinic acid could reduce the elevated level of blood urea N2, serum creatinine, renal MDA and immune expression autophagic marker protein (LC3/B), proapoptotic protein (Bax), autophagic marker protein (LC3/B), iNOS and autophagic marker protein (LC3/B). They also increased lower SOD level, GSH, GPx, and antiapoptotic protein (Bcl-2) immunoexpression [112]. In addition, previous researchers found that lycopene (10 mg/kg) attenuated fluoride toxicity via decreasing caspase-3 and caspase-9, and Bax gene expression functions, while improving the activities of GPX and SOD and Bcl-2 gene expression in rats. Ferrous ascorbate administration decreased spermatozoa mobility, viability and spermatozoa antioxidant capacity. It enhanced superoxide production, ROS generation and lipid peroxidation (ferrous ascorbate (FeAA)). On the other hand, the administration of lycopene prevented these changes and has significant antioxidant properties and ROS-scavenging activity in the case of male reproductive cells [113].

It is also able to reduce Aβ-induced oxidative stress due to the lower production of intracellular ROS and superoxide obtained by mitochondria. Moreover, it showed improved Aβ-related mitochondrial morphological modifications, releasing cytochrome-c and opening of mitochondrial transition pores. Lycopene was also found to be effective in restoring ATP in Aβ-treated neurons and improved mitochondrial complex activities. In vivo studies also revealed that in mitochondria, lycopene significantly prevented DNA damage and enhanced transcription factor A level [114].

### 4.5. Anti-Inflammatory Activity

Lycopene not only quenches singlet ROS, but also prevents lipid peroxidation, too. It has been shown that lycopene (50 and 100 μM) upregulated the HO-1 mRNA. However, it cannot regulate the *NOS2* mRNA and COX-2 expression. Such concentrations also suppressed the LPS-stimulated COX-2, *NOS2* and TNF-α gene expression in RAW264.7 cells [66]. Moreover, lycopene has a protective role in β-amyloid-induced inflammation. β-amyloid increased the serum IL-1β, TNF-α, IL-6β levels, and upregulated the expressions of NF-κB p65 mRNA, TLR4 and protein at the choroid plexus. Lycopene supplementation decreased the inflammatory cytokines and reversed the Aβ1-42-related expression and up-regulation of NF-κB p65 mRNA, TLR4 and protein at the choroid plexus [67].

Lycopene supplementation (12.5 mg/kg BW) in carrageenan-induced inflammation considerably decreased edema in Swiss mice via various phlogistic compounds and immunostaining for COX-2, iNOS and NF-κB. It also decreased the migration of leukocytes in paw tissue and peritoneal cavity, lowered the concentration of MPO, while increasing the GSH levels [68]. Furthermore, lycopene has been investigated in different doses (i.e., 0.5, 1.0, 2.0, 4.0, 8.0, 10.0 and 25 μM) for the prevention of cigarette smoking-induced inflammation. Lycopene inhibited the increase in interferon-γ, TNF-α and interleukin-10 concentrations [115]. In a recent study, induction of endotoxin-induced uveitis was performed in Sprague–Dawley rats by LPS single injection (200 μg). The lycopene intraperitoneal administration (10 mg/kg) noticeably lowered the elevated levels of infiltrating cell number, total protein concentration, and NO, TNF-α, and IL-6 induced via LPS [116].

Lycopene is also effective against the treatment of mitochondrial dysfunctioning caused by Aβ1-42 accompanied by TGF-β, increased proinflammatory cytokines, TNF-α and IL-1β and also NF-κB and caspase-3 function in the brain of rats [117]. Lycopene from tomato juice significantly lowered the ICAM-1, and vascular cell adhesion molecule 1 (VCAM-1) level [118]. Lycopene also protects rats against acute lung injury due to LPS by reducing the IL-6 and TNF-α level, suppressing MAPK activity and NF-κB transcription factor. As a result, normal metabolism is retrieved [119]. In LPS-challenge inflammation and depression, lycopene treatment (60 mg/kg/day, orally) for 7 days and also an intraperitoneal injection of LPS (1 mg/kg) was effective. In addition, lycopene improved neuronal cell damage in the hippocampal CA1 region. It reduced HO-1 and IL-1β LPS-related expression in the hippocampus as well as reducing TNF-α and IL-6 in the plasma [120,121].

In the case of THP1 cells, lycopene (2 μM for 2 h) strengthened pro-inflammatory gene expression. The effectiveness of lycopene dose is associated with using a pro-inflammatory stimulus (i.e., LPS, PMA or TNF). At higher concentrations (>2 Μm), lycopene improved metalloprotease secretion, which is a c-AMP-dependent process, by reducing the production of ROS. Cell culture media, PMA-treated monocytes and moved on CaCo-2 epithelial cells could induce proinflammation in these cells. Such inflammation was overcome by treating the cells with lycopene within 12 h. At a lower level of lycopene (<2 μM), it promotes an inflammatory condition not associated with the modulation of ROS. When the concentration of lycopene increases (5 to 20 μM), it was noted that ROS production decreases and consequently, an antiinflammatory effect was produced [122].

### 4.6. Hepatoprotective

Functional mitochondria perturbation is related to fulminant hepatic failure. Recently, in a study conducted on animal models, d-GalN/LPS (dose: 300 mg/kg and 30 μg/kg BW) induced several instabilities in mitochondrial functioning by increasing H_2_O_2_ and lipid peroxide levels, decreasing the antioxidant activities of mitochondria, disturbing enzymatic functions of the electron transport chain, tricarboxylic acid cycle and adenosine triphosphate content in cells. Lycopene (10 mg/kg BW, through 6 days) reduced lipid peroxidation and limited excessive H_2_O_2_ production. Lycopene consumption also regulated d-GalN/LPS-related disturbance in ATP synthesis and elevated enzymatic functions [123]. In addition, pre-treatment with lycopene (5, 10, and 20 mg/kg) has a protective role in a rat model of non-alcoholic fatty liver disease through lowering the liver enzymes levels, like aspartate transaminase (AST), alanine transaminase (ALT), LDL, free fatty acid, and MDA. Furthermore, its role was conducted via an increase in the SOD and GSH concentrations in liver tissue, down-regulating the CYP2E1 and TNF-α expression and reducing the penetration of liver fats [69]. In another study by Yefsah-Idres et al. (2016), it was shown that lycopene administration ameliorated liver injury. It decreased the levels of ALT, serum homocysteine, and AST. In addition, its hepatoprotective effect was performed by enhancing the S-adenosyl-homocysteine hydrolase, hepatic cystathionine beta-synthase activities and decreasing the level of hepatic MDA [70]. Moreover, tomato powder has been shown to have a protective agent against alcohol-induced hepatic injury by inducing cytochrome p450 2E1 [80]. The lycopene treatment considerably improved liver functioning in case of rats with bile duct ligation (BDL). It reduced NO and MDA levels and improved reduced enzymatic level (i.e., CAT, GSTs, GSH, and SOD) in the BDL rat. In addition, lycopene decreased DNA damage [71].

The efficacy of lycopene (40 mg/kg) with proanthocyanidins (450 mg/kg) against mercuric chloride-induced hepatotoxicity was assessed in an animal model. It has been revealed that they inhibited ROS production, protected antioxidant enzymes, and reversed hepatotoxicity in rats’ liver [124]. In another study, carbon tetrachloride-caused hepatotoxicity was done in male Wistar rats via intraperitoneal injection (dose; 0.1 mL/kg BW, 14 days). Then, the *Portulaca oleracea* aqueous extract with lycopene (dose; 50 mg/kg body weight) exhibited a hepatoprotective effect against carbon tetrachloride-induced hepatotoxicity. The results also reveal that lycopene significantly restores serum enzyme level to the normal amount in rats [125].

In a study on methotrexate-induced liver injury (20 mg/kg), histopathologic examination of the rats showed that inflammatory cell infiltration, sinusoidal dilatation and congestion were improved significantly by lycopene consumption (10 mg/kg). According to the results, IL-1β, and TNF-α concentrations in the liver tissue were reduced significantly compared to the control group. At the same time, a reduction in oxidative stress index (OSI) was non-significant [126]. In another study on acetaminophen-induced liver damage in C57BL/6 mice, lycopene treatment improved redox imbalance, decreased IL-1β expression, thiobarbituric acid reactive species level and MMP-2 activity [127]. Another study on the hepatoprotective role of lycopene focused on ATZ-induced hepatotoxicity. ATZ (50 and 200 mg/kg) induced hepatotoxicity, whereas lycopene (5 mg/kg) significantly reduced the total cytochrome b5 (Cyt b5) and CYP450 contents. In addition, it prevented from CYP450s stimulation activity (erythromycin *N*-demethylase (ERND), aniline-4-hydroxylase (AH), aminopyrine *N*-demethylase (APND), and NADPH-cytochrome c reductase (NCR)) in microsomes of the liver. Lycopene administration effectively modulates CYP450s activities and contents and also normalizes the four CYP450s genes expression, including CYP2a4, CYP2E1, CYP1b1, and 4A14 [128,129].

Furthermore, lycopene (10 mg/kg BW/day, intraperitoneal administration) markedly prevented oxidative damage in experimental hepatitis due to d-galactosamine/LPS. It improved the enzymatic antioxidants levels (i.e., CAT, GPx, SOD, and GSTs) as well as non-enzymatic antioxidants (i.e., vitamin C and E, and GHS). Moreover, lycopene reduced the high level of lipid peroxides and decreased the DNA strand breaks [130]. Additionally, d-Gal/LPS could induce hepatitis in experimental rats, and lycopene significantly reduced the lipid metabolizing enzymatic activity (i.e., LPL, lecithin-cholesterol acyltransferase (LCAT), hepatic triglyceride lipase (HTGL), and increase in the HDL level [131]).

### 4.7. Against Dermatologic Diseases

Treatment with lycopene decreased UVB-caused cell proliferation while increasing apoptosis via declining CDK2 and CDK4 in hairless SKH-1 mice and human keratinocytes [82]. Moreover, the study conducted on volunteers revealed that lycopene decreased the expression of UVA1 radiation-inducible genes HO-1 upregulation caused by UVA1 and UVA/B [72].

### 4.8. Neuroprotective 

The lycopene consumption relieved cognitive defects, age-related memory loss, neuronal damage, and synaptic dysfunction of the brain. Furthermore, lycopene consumption considerably reduced age-related neuroinflammatory disorders by decreasing microgliosis (IBA-1), as well as down-regulating inflammatory mediators. In addition, a study revealed that lycopene down-regulates the Aβ1-42 accumulation in the brains of aged CD-1 mice [62]. Similarly, lycopene (0.03% *w*/*w*) has been found to inhibit the LPS (0.25 mg/kg)-induced memory loss in 3-month old male C57BL/6J mice by reducing Aβ accumulation, APP, reducing the expression of neuronal β-secretase beta-site amyloid precursor protein cleaving enzyme 1 (BACE1), and improving the α-secretase A disintegrin and metalloproteinase 10 (ADAM10) expressions. In addition, it suppressed IBA-1 expression as the marker of microglia activation, inflammatory mediators and also decreased oxidative stress (among mice treated with LPS). Furthermore, lycopene inhibited NF-κB, MAPKs and Nrf2 activity phosphorylation in BV2 microglial cells (LPS treated) [63].

Lycopene suppressed the 4-AP-invoked release of glutamate and elevated intra-synaptosomal Ca^2+^ level. The studies revealed that the inhibitory impact of lycopene on 4-AP-evoked glutamate secretion decreased the presence of Cav2.1 (P/Q-type) and Cav2.2 (*N*-type) channel blocker ω-conotoxin MVIIC noticeably. However, it did not affect the inhibitors of CGP37157 and intracellular Ca^2+^ release. Moreover, lycopene’s effect on evoked glutamate secretion was protected via PKC inhibitors Go6976 and GF109203X [73]. Wang et al. reported that rats that were fed on HFD with lycopene (4 mg/kg, oral administration) had considerably reduced cognitive defects and improved learning abilities through the prevention of a decrease in dendritic spine density [132]. In another study, lycopene administration (5, 10 mg/kg, oral) significantly decreased impairment in biochemical, behavioral, neuroinflammatory and neurochemical markers in rats with haloperidol-induced orofacial dyskinesia [75].

Lycopene’s neuroprotective effect was also studied for pentylenetetrazol (PTZ)-induced kindling epilepsy in rats treated with lycopene at the concentrations of 2.5, 5, and 10 mg/kg, sodium valproate through 29 days, and 40 mg/kg of PTZ (intraperitoneal) with alternate days. Rats were assessed for various behavioral and biochemical parameters (SOD, lipid peroxidation, CAT, decreased GSH and nitrite) and activities of the enzymes of mitochondria (I, II, and IV) in the brain. Based on the findings, lycopene (5, 10 mg/kg) considerably improved mitochondrial enzymatic activities, kindling score, and oxidative stress as compared to control [74]. The neuroprotective role of lycopene was also studied in a male C57BL/6 mice model of bilateral common carotid artery occlusion (BCCAO). The Nrf2/HO 1 signaling route function via increasing the Nrf2 HO 1 expression was also seen. The results indicate that the Nrf2/HO 1 signaling route is associated with lycopene’s neuroprotective impact [133].

Regarding AD, lycopene considerably prevents paralysis in Aβ1-42-transgenic *Caenorhabditis elegans* strain GMC101. Treating with lycopene inhibited Aβ1-42 release in SH-SY5Y cells by the overexpression of the Swedish mutant type of human APP (APPsw). Additionally, lycopene can effectively down-regulate APP expression in APPsw cells. Furthermore, treating with lycopene did not affect endogenous ROS amount and apoptosis in case of APPsw cells. It significantly improved the neurological scores in BCCAO mouse. It reduced neuronal apoptosis via TUNEL staining and decreased oxidative stress due to global brain ischemia [134]. Interestingly, it has a neuroprotective effect against PD caused by 1-methyl-4-phenyl-1,2,3,6-tetrahydropyridine (MPTP) in mice. Treating with lycopene (5, 10 and 20 mg/kg/day, oral administration) prevented PD by MPTP in a concentration-dependent fashion. It also reduced MPTP-caused motor abnormalities and oxidative stress [135]. Chronic consumption of lycopene can effectively improve cognitive abilities, decrease mitochondrial-oxidative damage, inhibit neuroinflammation and repair BDNF concentration in β-A1-42-treated rats [136].

The lycopene neuroprotective effect on oxidative stress, as well as neurobehavioral disorders in PD of adult C57BL/6 mice due to rotenone, was studied in a recent study. Lycopene treatment (10 mg/kg BW, oral administration) in the rotenone-treated animals enhanced GSH-Px, SOD and CAT activities and reduced MDA concentrations. It also elevated the number of tyrosine hydroxylase-, enhanced alpha-synuclein (alpha-SYN)- and microtubule-associated protein 3 light chain (LC3-B)-positive neurons [137]. Lycopene provides protection against neurotoxicity through methylmercury (MeHg) induced in cultured rat cerebellar granule neurons (CGNs). Lycopene prevented the loss of cell viability and release of LDH. Lycopene also protected from the inhibition of mitochondrial complexes III and IV enzyme activities and decreased the production of ATP and mtDNA (copy, transcript levels) [138].

There is an in vivo study on rat’s treatment using rotenone (3 mg/kg BW, intraperitoneal) for 30 days. NADH dehydrogenase as a marker of rotenone action was markedly suppressed (35%) in treated animals’ striatum. On the other hand, oral administration of lycopene (10 mg/kg) to the rotenone-treated animals for 30 days elevated the activity by 39% compared to the animals treated with rotenone. Rotenone treatment enhanced the MDA concentrations (75.15%) in the striatum, while lycopene treatment reduced its concentrations by 24.33%. This was associated with cognitive and motor impairments in rotenone-treated animals that were reversed on lycopene administration. Finally, lycopene administration inhibited the cytochrome c release from mitochondria [139].

### 4.9. Bone Protective

Lycopene has several molecular and cellular effects on human osteoblasts and osteoclasts. It reduced osteoclast differentiation, whereas it did not change cell survival/cell density; calcium-phosphate resorbing was also reduced. In addition, osteoblast proliferation (reduction on apoptosis) and differentiation were improved by lycopene [76,140]. Moreover, lycopene supplementation has an effective role in postmenopausal osteoporosis [80]. Lycopene administration inhibited the ovariectomized (OVX)-related increase in bone turnover, such as modification in biomarkers of serum osteocalcin, serum cross-linked carboxyterminal telopeptides, bone metabolism, serum *N*-terminal propeptide of type 1 collagen, and urinary deoxypyridinoline. Remarkable upgrading in OVX-related loss of bone mass, microarchitectural deterioration and bone strength was witnessed in lycopene-administrated OVX animals. These observations were more prominent in areas full of trabecular bone and low level of cortical bone. In these six-week-old Sprague–Dawley female rats, the bone mineral density of the tibial proximal metaphysis and lumbar spine increased via lycopene treatment dependent on the dose. Lycopene administration also down-regulated the osteoclast differentiation along with up-regulation of the osteoblast and GPx CAT and SOD activities [77,141]. Daily lycopene consumption reduces the risk of bone resorption in postmenopausal female cases by oxidation prevention [142].

### 4.10. Targeting Reproductive Disorders

Lycopene can decrease sperm DNA fragmentation, as well as lipid peroxidation by its antioxidant activity in normospermic infertile men [78,105]. It (4 mg/kg/day, orally) improved the sperm count and motility by decreasing H_2_O_2_ and lipid peroxidation, and improving mitochondrial enzymatic activity (i.e., CAT, SOD, GR, GPx, and ADH) and non-enzymatic antioxidant level (GSH and ascorbate). In addition, lycopene showed a significant improvement in the testicular mitochondria’s enzymatic activities of tricarboxylic (i.e., isocitrate dehydrogenase, succinate dehydrogenase, fumarase and malate dehydrogenase) [79].

Lycopene (4 mg/kg) ameliorated adriamycin (ADR) (10 mg/kg)-induced reductions in Sprague–Dawley rats’ epididymis and testes weights. The sperm morphology and motility were noticeably normalized by lycopene treatment. While the testosterone amount was reduced in the ADR group, no significant effect was found in the treated group. Interestingly, pretreatment with lycopene significantly reformed MDA and decreased GSH levels. Moreover, ADR-induced histopathological changes were reversed by the lycopene treatment [143].

**Figure 2:**
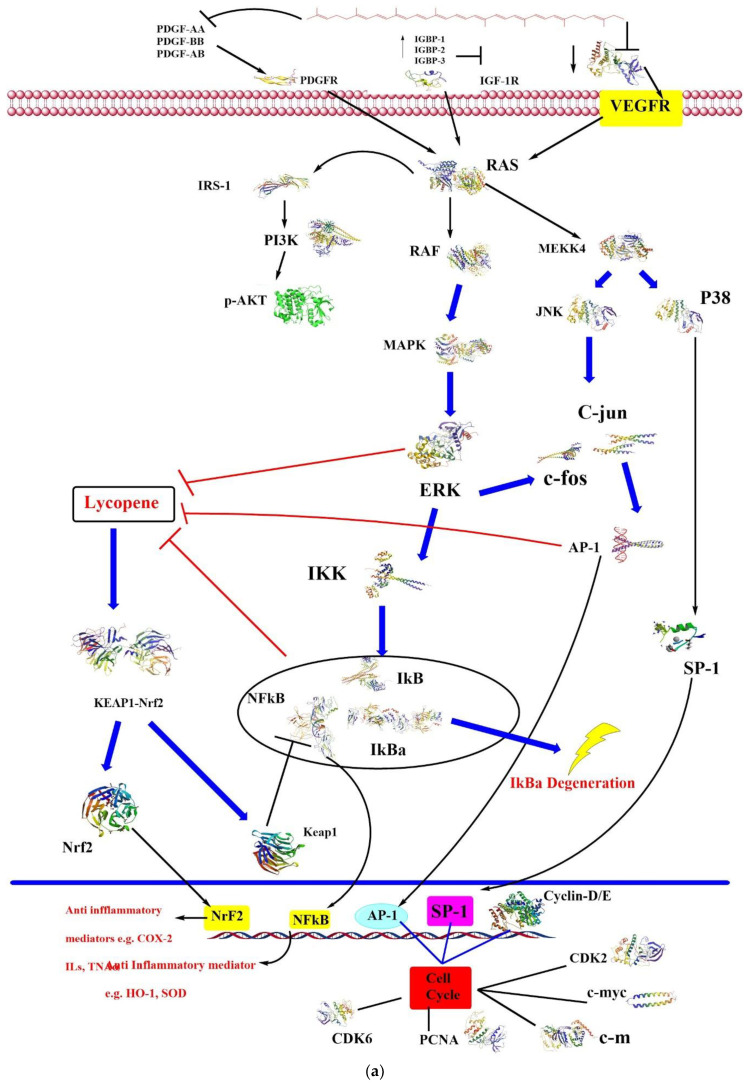

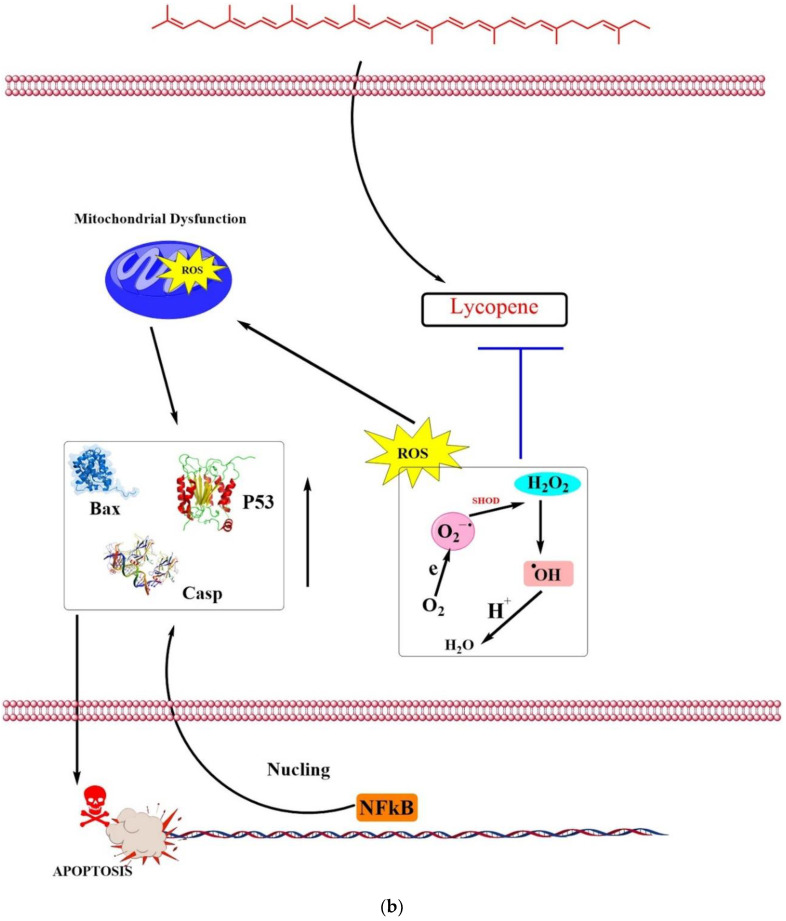
(**a**). Effect of lycopene on PDGFR-, IGF-IR-, and VEGFR-mediated signal pathways. (**b**) Effect of Lycopene on Reactive Oxygen Species (modified from [146]).

Lycopene has protective effects against cisplatin-induced spermiotoxicity, too. Lycopene (4 mg/kg) showed significant results in decreasing total abnormal sperms, improving sperm viability and motility. In addition, lycopene decreased testicular MDA concentration and increased GPx activity [144]. Another study revealed a protective effect of lycopene (1.5 mg/0.5 mL Tween; 80/100 g BW) in cyproterone acetate-induced infertility in rats. Lycopene made a significant retrieval effect on sperm count, viability, and motility, hypo-osmotic swelling tail to coil spermatozoa, testicular functions of 17β- hydroxysteroid dehydrogenase (HSD), CAT, 3β-HSD and SOD, conjugated diene level, MDA, serum testosterone, and testicular cholesterol [145]. Figure 2 illustrates some of the common signaling pathways which are mentioned in this section. The figure was designed and modified based on Trejo-Solís and co-authors’ paper [146].

VEGF: vascular endothelial growth factor; AP-1: activator protein-1; CASP: caspase; ERK1/2: kinase regulated by extracellular signals; IGF: insulin-like growth factor; IKK: IκB kinase; IRS: insulin-1 receptor substrate; Keap1: Kelch-like ECH-associated protein 1; PCNA: proliferating cell nuclear antigen; PDGF: platelet-derived growth factor; PI3K: phosphoinositide 3-kinase; RAF: proto-oncogene serine/threonine-protein kinase; SP-1: specificity protein 1.

## 5. Protective Effects of Lycopene Against Different Toxins

Besides the aforementioned anti-toxicity effects of lycopene, this action should be briefly discussed. Previous studies suggested its significant protection against a wide variety of natural and chemical toxins. Different chemical agents with known neurotoxicity, hepatotoxicity, nephrotoxicity, and cardiotoxicity are inhibited by lycopene. Lycopene’s main properties which are believed to be necessary for this action are anti-oxidative, chelating, free-radical scavenging and antiapoptotic activities. This miracle pigment prevents bacterial toxins, mycotoxins, fluoride, metals, and pesticides from exerting their toxicities against the human body [4].

## 6. Recommended Dose

It should be noted that there is no ideal dose for daily lycopene consumption. Although previous studies only give ideas about lycopene intake at a different level, that would be helpful. For example, an in vivo study revealed that lycopene (6.5 mg/day) was effective against cancer in men [147]. However, lycopene dose should be increased up to 10 mg/day, in the case of advanced PCa. In another study, lycopene supplementation (15 mg/day, for 12 weeks) in an old aged population improved immune function through increasing natural killer cell activity by 28% [148]. Therefore, it seems that different lycopene doses and duration of supplementation can be suggested for various health purposes. Finally, according to different epidemiological studies, daily lycopene intake can be suggested to be 2 to 20 mg per day [10].

## 7. Safety and Toxicity

Safety monitoring should be considered as an important issue about medicinal plants and plant-derived phytochemicals [149,150]. There are several in vitro and in vivo studies on the possible toxicity of lycopene. For instance, it has been shown that lycopene (up to 10 μM) has no toxicity on the rat’s cultured cerebellar granule neurons viability [138]. Another research on cultured rat hippocampal neurons showed no significant toxic effects of lycopene when it was applied to these cells [151], even though it seems that carotenoids in some certain settings at high tissue concentrations may show a pro-oxidant effect [152]. A toxicological study on rats showed the no-observed-adverse-effect level at the highest examined dose (i.e., 1.0% in the diet) [153]. It should be noted that different lycopene forms (i.e., lycopene extracted from tomato, synthetic lycopene, and its crystallized extract) are generally recognized as safe when used in different food products [154]. There are no reports on adverse events from lycopene use at normal and ordinary doses [155]. Human studies suggested the no-observed-adverse-effect level for lycopene as 3 g per day/kg of body weight. Daily lycopene intake has been estimated as being significantly less than this level. Even the 99th percentile for its intake is 123 mg per day [156]. It seems that the expression of cytochrome P450 2E1 can be induced by high doses of lycopene and alcohol. Therefore, their high dose of concomitant use should be avoided [157]. In addition, regarding lycopene’s potent antioxidative effect, precautions should be kept in mind for patients under chemo and radiation therapy [158]. A case report described lycopenemia in a woman who had ingested about 2 liters of tomato juice every day and for several years. She had lycopene deposits in the liver (with no evidence of hepatic dysfunction) and deep orange discoloration of the skin. The lycopenodermia disappeared 3 weeks after stopping tomato juice consumption [159]. In addition, there is a chain of studies in which the lycopene mutagenicity was assessed. The results indicate that formulated lycopene has no mutagenic effects, although crystalline lycopene showed some mutagenic activity when degraded under exposure to light and air [160].

## 8. Conclusions

Lycopene possesses potent anticancer, antioxidant, antiinflammatory, and antidiabetic potential. In addition, it is a nutraceutical which protects against a wide variety of heart, liver, bone, skin, nervous, and reproductive systems diseases, as evident from numerous studies. However, further investigations are necessary to unveil the underlying mechanisms of actions, with a special emphasis on gene expression studies. Additionally, the recommended and effective doses of this functional food need to be further investigated. Safety concerns about its genotoxicity, maternal toxicity, and teratogenic effects should also be inquired.

## Figures and Tables

**Table 1:** Lycopene content in different sources.

Food Sources	Contents (mg/100 g)
Fresh tomatoes	0.72–4.2
Cooked tomatoes	3.70
Tomato sauce	6.20
Tomato paste	5.40–150
Ketchup	9.90–13.44
Pumpkin	0.38–0.46
Sweet potato	0.02–0.11
Pink grapefruit	0.35–3.36
Carrot	0.65–0.78
Pink guava	5.23–5.5
Watermelon	2.30–7.20
Apricot	0.01–0.05
Papaya	0.11–5.3
Rosehip	0.68–0.71

**Table 2:** Various mechanisms for lycopene’s biological effects.

Biological Effect	Mechanisms of Action	References
Anticancer	Reduced nuclear factor-kappa B (NF-κB) expression, serum level of oxidative stress marker malondialdehyde (MDA) Improved nuclear factor erythroid 2 expressions Reduced the expression of signal transducer and activator of transcription 3 (STAT3) Induced the protein inhibitor of activated STAT3 expression	[44,45]
	Induced cell cycle arrest and modified the potential of mitochondrial membrane, DNA fragmentation	[46,47]
	Treatment of HGC-27 cells with lycopene exhibited significant improvement in LC3-I, Phosphorylated Extracellular Signal-Regulated Kinase (p-ERK) proteins expressions	[48]
	Reduced NADPH oxidase (NOX) 4 activity Lowered invasion, migration, and adhesion, NOX activity, matrix metalloproteinase (MMP)-9, MMP-2 activities and NOX4 protein expression	[49]
	Reduced metastatic load and inhibited cancer antigen 125 (CA125) expression Down-regulated expression of Integrin beta-1 (ITGB1), MMP9, Integrin alpha-5 (ITGA5), Focal Adhesion Kinase (FAK), integrin-linked kinase (ILK) and EMT markers Decreased activity of mitogen-activated protein kinase (MAPK) and inhibited integrin α5 protein expression	[50]
Antidiabetic	Lowered MDA levels, and increased antioxidant enzyme activities	[51]
	Decreased the glycated haemoglobin (HbA1c) and C-reactive protein (CRP)	[52,53]
	Enhanced antioxidant enzymes activities (i.e., superoxide dismutase (SOD), glutathione peroxidase (GPx), catalase (CAT), glutathione-S-transferase)	[54]
	Lowered the expression of BCL2-associated X protein (BAX), but improved B-cell lymphoma-extra large (Bcl-xL) levels and B-cell lymphoma 2 (Bcl-2)	[55]
	Downregulated RAGE expression and declined NF-κB and MMP-2 expressions	[56]
	Reduced levels of serum nitrate-nitrite level	[57]
Cardioprotective	Decreased low-density lipoprotein-cholesterol (LDL), total cholesterol (TC), and thiobarbituric acid-reacting substances	[58]
	Lowered very-low-density lipoprotein-cholesterol (VLDL), triglycerides (TG) and increased high-density lipoprotein-cholesterol (HDL) level	[59]
	Decreased inflammatory factors (CRP, interleukin (IL)-6), pulse wave velocity, adhesion molecules and endothelial function	[60]
	Lowered the expression of Rho-associated protein kinase (ROCK)1, Ki-67, intercellular adhesion molecule-1 (ICAM-1) and ROCK2 Improved the expression of endothelial nitric oxide synthase (eNOS) implanted arteries and cGMP plasma concentration	[61]
Antioxidant	Increased CAT, glutathione (GSH) and SOD activities and mRNAs antioxidant enzyme Reduced age-related neuroinflammatory disorders by decreasing microgliosis (Ionized calcium-binding adaptor protein-1 (IBA-1)) Down regulate the accumulation of amyloid beta (Aβ) _1__-42_ in the old CD-1 mice’s brain.	[62]
	Decreased Aβ accumulation, amyloid precursor protein (APP) Reduced the expression of neuronal β-secretase (BACE)1 Improved the expressions of α-secretase A Disintegrin And Metalloproteinase (ADAM)10 Suppressed IBA-1 (a marker of microglial activation) expression, inflammatory mediators Inhibited NF-κB, phosphorylation of MAPKs and Nuclear factor erythroid 2-related factor 2 (Nrf2) activity	[63]
	Decreased myeloperoxidase (MPO) activity, tumor necrosis factor-alpha (TNF-α) Down-regulated gene expression of inducible nitric oxide synthase (iNOS)	[64]
	Decreased inflammatory cytokines Improved antioxidant enzymes NAD(P)H: quinone oxidoreductase (NQO1) and heme oxygenase-1 (HO-1) mRNA expressions Downregulated inflammatory cytokines TNF-α and IL-1β Improved the glial cells inflammatory markers glial fibrillary acidic protein (GFAP) and IBA-1 expression	[65]
Antiinflammatory	Up-regulated the HO-1 mRNA Gene expression suppression of the TNF-α and lipopolysaccharide (LPS)-stimulated cyclooxygenase-2 (*COX-2*), nitric oxide synthase 2 (*NOS2*)	[66]
	Lowered the IL-1β, TNF-α, IL-6β serum level and increased the NF-κB p65 mRNA, Toll-like receptor 4 (TLR4) and protein expressions	[67]
	Inhibited COX-2, iNOS and NF-κB Reduced the leukocytes migration	[68]
Hepatoprotective	Lowered aspartate aminotransferase, alanine aminotransferase, free fatty acid, MDA and LDL Increased the SOD, GSH condensation Down-regulated the expression of Cytochrome P450 2E1 (CYP2E1), TNF-α	[69]
	Enhanced the S-adenosyl-homocysteine hydrolase, hepatic cystathionine beta-synthase activities	[70]
	Reduced nitric oxide (NO) and MDA levels	[71]
Against dermatologic diseases	Decreased Ultraviolet (UV) B-induced cell growth and increased apoptosis Prevented from Forkhead box O3 (FOXO3) phosphorylation when exposing to UVB radiation and cytoplasm sequestering Decreased cyclin-dependent kinase 4 (CDK4) and CDK2 Increased in the expression of Poly (ADP-ribose) polymerase (PARP), BAX and cell apoptosis Inhibited UVA1- and UVA/B-induced upregulation of HO-1	[72]
Neuroprotective	Suppressing 4 aminopyridines (4-AP) evoked glutamate release and elevated intrasynaptosomal Ca^2+^ level Suppressing release of 4-AP-evoked glutamate	[73]
	Improved mitochondrial enzymatic activities, kindling score, oxidative stress	[74]
	Decreased impairment in biochemical, behavioral, neuroinflammatory and neurochemical markers	[75]
Boneprotective	Osteoblasts differentiation improved	[76]
	Modified the biomarkers of serum osteocalcin, bone metabolism, crosslinked carboxyterminal telopeptides and *N*-terminal propeptide of type 1 collagen in serum Downregulated the osteoclast differentiation concurrent Up-regulated the osteoblasts alongside GPx, SOD, and CAT activities	[77]
Targeting reproductive disorders	Reduced lipid peroxidation and sperm DNA fragmentation	[78]
	Improved the sperm motility and sperm count Improved mitochondrial enzymatic activity, i.e., CAT, SOD, glucocorticoid receptor (GR), GPx and alcohol dehydrogenase (ADH)) and non-enzymatic antioxidant level (GSH and ascorbate)	[79]
